# The influence of blood lipids on cerebral perfusion by apolipoprotein E status

**DOI:** 10.1016/j.jlr.2026.101057

**Published:** 2026-05-14

**Authors:** Kevin P. Decker, Nicholas A. Rizzi, Zoe Rigas, Catherine Awad, Elizabeth M. Habash, Mary K. Kramer, Alexander M. Cerjanic, Matthew L. Cohen, Curtis L. Johnson, Christopher R. Martens

**Affiliations:** 1 Department of Kinesiology and Applied Physiology, University of Delaware, Newark, DE, USA; 2 Department of Biomedical Engineering, University of Delaware, Newark, DE, USA; 3 Department of Neurology, Massachusetts General Hospital, Boston, MA, USA; 4 Department of Communication Sciences & Disorders, University of Delaware, Newark, DE, USA

**Keywords:** apolipoprotein E, cerebral blood flow, hippocampus, lipids, and triglycerides

## Abstract

Elevated blood lipids are strongly associated with increased risk of dementia due to Alzheimer's disease (AD). The apolipoprotein E (*APOE*) gene plays an important role in lipid transport and is a known genetic risk factor for the development of AD, with increased risk in ε4 carriers. Reduced cerebral blood flow (CBF) is known to precede the onset of AD pathology; however, the associations between blood lipids and cerebral perfusion by *APOE* status are not completely understood. This study included 65 midlife and older adults (≥50 years old), of which 18 (28%) carried the *APOE* ε4 allele. Using arterial spin labeling, we measured gray matter and white matter (WM) CBF, and hippocampal blood flow. Pearson correlations and simple linear regressions were used to assess the associations between blood lipids and cerebral perfusion. Serum triglycerides and very-low density lipoprotein cholesterol were negatively associated with GM CBF, WM CBF, and hippocampal blood flow in all participants. However, when stratified by *APOE* status, the negative associations of triglycerides and very-low density lipoprotein cholesterol on cerebral perfusion were more pronounced in ε4 carriers than non-ε4 carriers, despite no significant group differences in blood lipids. High-density lipoprotein cholesterol was positively associated with only WM CBF, without any differences between *APOE* status. No other blood lipids were associated with resting cerebral perfusion. These findings suggest that blood lipids can influence resting cerebral perfusion, and ε4 carriers are more negatively affected by this association which may partially explain the increased genetic risk for AD pathology.

Aging is a well-established primary risk factor for cognitive decline and the development of Alzheimer's disease (AD) (1). AD currently affects nearly 6.9 million Americans over the age of 65 years old (2) as the population increasingly reflects a demographic shift of older adults, the number of AD cases are projected to double over the next 20 years (2), and is rapidly becoming a public health concern (https://www.alzint.org/about/dementia-facts-figures/dementia-statistics/). Living with AD imposes significant financial and emotional burdens on patients, caregivers, and the healthcare system. Recently approved therapies for early AD effectively remove amyloid beta plaques but only modestly slow cognitive decline and cause side effects in up to a third of patients (3, 4). Accordingly, improved understanding of the risk factors and biological mechanisms driving AD is needed to develop effective strategies for disease prevention.

Adopting a healthy lifestyle is among the best options for primary and secondary prevention of AD, with recent evidence suggesting that up to 45% of the worldwide dementia cases may be prevented by addressing a series of modifiable risk factors (5). Elevated low-density lipoprotein cholesterol (LDL-C) is among these risk factors and could lower the number of cases of dementia by up to 7% if properly addressed. However, the mechanisms linking elevated LDL-C to dementia risk are not completely understood. Moreover, the link between elevated LDL-C and dementia risk may be exacerbated by nonmodifiable risk factors such as apolipoprotein E (*APOE*) genotype (6, 7). The *APOE* gene encodes for a component of lipoproteins involved in lipid transport (8). Polymorphisms in the *APOE* gene expressing the ε4 variant, as opposed to the ε2 or ε3 variant, have an increased risk of late-onset AD (9). Further, carriers of the ε4 variant are associated with higher blood LDL-C and triglycerides (TGs) (10, 11, 12).

Age-related increases in LDL-C and TG typically peak or plateau in the 5^th^ or 6^th^ decade of life, otherwise known as midlife (13, 14) and contribute to impaired vascular function and perfusion of vital organs (15, 16), which may be an important mechanism by which the ε4 allele increases AD risk. Resting cerebral blood flow (CBF) declines during midlife (17, 18) and cerebral hypoperfusion has been linked to age-related neural dysfunction that precedes AD (19, 20, 21, 22). One of the first brain regions to be affected by impaired CBF is the hippocampus (23), which plays a central role in learning and memory. Age-related reductions in global and regional cerebral perfusion have been associated with the *APOE* ε4 genotype (24, 25) and may be partially explained by cardiometabolic risk factors, including elevated LDL-C (24).

In this context, we aimed to test the associations between blood lipids and resting cerebral perfusion and their interactions with *APOE* genotype in midlife adults. We hypothesized that TG, total cholesterol, non-HDL-C, LDL-C, and very-low density lipoprotein cholesterol (VLDL-C) would be negatively associated with resting whole brain and hippocampal blood flow, while the cardioprotective high-density lipoprotein (HDL-C) would be positively associated with the same measures. We also hypothesized that the *APOE* genotype would influence the strength of these associations, with more pronounced associations in ε4 carriers than in non-ε4 carriers.

## Materials and Methods

### Study participants

Midlife and older adults (≥50 years old) were recruited from Newark, Delaware and surrounding areas. All participants were considered generally healthy, defined as nonobese, nonsmokers, and without any chronic diseases. None of the participants were actively taking medications or supplements known to lower blood pressure (e.g., beta-blockers, ACE inhibitors, etc.) or blood lipids (e.g., statins, fibrates, etc.). Participants were excluded if unable or unwilling to participate in an MRI scan (e.g., metal implants, claustrophobia, etc.). All female participants were at least one year post menopause and not receiving any hormone replacement therapy. The study was approved by the University of Delaware’s Institutional Review Board and all experimental testing conformed to the standards outlined in the Declaration of Helsinki. Informed consent was obtained from all participants prior to beginning data collections.

### Study visit

Participants reported to the laboratory in the morning (07:00-09:00) after an overnight fast (≥8 h) and refrained from alcohol and vigorous exercise for at least 24 hours and over-the-counter medications for at least 48 hours. Height and weight were measured, and blood was collected prior to the MRI brain scan. Afterward, participants underwent a brief cognitive assessment.

### Blood collection

Blood was drawn from the antecubital vein into an 8.5 ml Vacutainer serum separator tube and inverted several times to interact with the spray-coated silica to accelerate the clotting process. The tube was maintained in the upright position for 30 min to allow clotting, then centrifuged at room temperature for 15 min at 1500 RCF prior to being sent to a commercial clinical laboratory (LabCorp) for a standard lipid panel (test number: 303,756). TG, total cholesterol, and HDL-C, were measured directly, but LDL-C and VLDL-C were calculated using the NIH-Sampson equations (26), as shown below.

$$LDL-C=\left(\;\frac{Total\;Cholesterol}{0.948}\;\right)-\left(\frac{HDL-C}{0.971}\right)-\left[\left(\frac{TG}{8.56}\right)+\left(\frac{TG\;x\;nonHDL-C}{2,140}\right)-\left(\frac{TG^{2}}{16,100}\right)\right]-9.44$$

$$VLDL-C=\left(\;\frac{TG}{8.59}\;\right)+\left(\frac{TG\;x\;nonHDL-C}{2,250}\right)-\left(\frac{{TG}^{2}}{16,500}\right)$$

Additional blood was drawn into an ethylenediaminetetraacetic acid-coated Vacutainer tube and inverted several times to accelerate the anticoagulation process. The noncentrifuged ethylenediaminetetraacetic acid tube was sent to LabCorp for *APOE* genotyping (test number: 504040) by PCR amplification of a specific region in exon 4 of the *APOE* gene followed by digestion with Hha I restriction enzyme and separation of fragments by polyacrylamide gel electrophoresis (27). This approach allows the *APOE* ε2, ε3, and ε4 alleles to be distinguished.

### Arterial spin labeling

MRI images were acquired using a 64-channel head coil in a 3T Siemens Prisma MRI scanner (Siemens). Participants lay in a supine position with a foam pad under their legs for comfort. High-spatial-resolution volumetric T1-weighted magnetization-prepared rapid acquisition gradient-echo anatomical images of the brain were acquired with the following parameters: repetition time (TR)/inversion time (TI)/echo time (TE) = 2,300/900/2.32 milliseconds (ms), spatial resolution = 0.9 × 0.9 × 0.9 mm^3^, 192 sagittal slices.

Gray matter (GM) and white matter (WM) CBF were acquired using the multi-PLD protocol from the Human Connectome Project, as previously described (17, 28, 29). The imaging parameters were as follows: TR/TE = 3,705/26.4 ms; spatial resolution = 2.5 × 2.5 × 2.3 mm^3^; 215 × 215 mm^2^ field-of-view; 86 × 86 matrix, 43 slices; 43 pairs of control/label images; total acquisition time = 5.5 min. The labeling duration was 1,500 ms with five PLDs = 200 ms (control/label pairs = 6), 700 ms (pairs = 6), 1,200 ms (pairs = 6), 1700 ms (pairs = 10), and 2,200 ms (pairs = 15). Signal readout was implemented using 2D multi-band gradient-echo EPI using partial Fourier = 6/8 and SMS acceleration factor = 1.

CBF maps were obtained with a standard kinetic-model inversion using the Bayesian algorithm under the graphic user interface BASIL in FSL (30). Two equilibrium echo magnetization (M0) images were acquired at the scans end and averaged to produce a calibration image. Perfusion maps were calibrated onto the proton density-weighted M0 image using voxel-wise calibration mode with a sequence TR = 8 s and a calibration gain = 1. CBF for each voxel was calculated using the following formula: where λ = 0.9 ml/g (brain/blood barrier partition coefficient); SI control and SI label = time-averaged signal intensities of control and label images; T 1b = 1.65 s (longitudinal relaxation time of blood); α = 0.85 (labeling efficiency); SI PD = signal intensity of proton density-weighted image; τ = labeling duration. The factor of 6,000 was used to convert the units from mL/g/s to mL/100 g/min (31). The segmentation of the hippocampus was performed with Freesurfer, allowing the quantification of hippocampal blood flow (HBF) (32).

### Cognitive assessment and AD biomarkers

Cognitive function was assessed using the Hopkins verbal learning test, which tests hippocampal-dependent memory encoding, recall, and recognition. Raw scores from the three immediate recall trials were summed to calculate immediate total recall. The immediate total recall and delay recall scores were converted into an age-calibrated T-score (M = 50, SD = 10) (33). Confirmation of AD pathology was not feasible due to the lack of Positron Emission Tomography imaging capabilities. However, biomarkers of AD pathology were measured retrospectively to characterize our cohort. Plasma pTau217 was assessed due to its strong agreement with amyloid Positron Emission Tomography imaging (34) using a Quanterix SR-X Biomarker Detection System. Further, white matter hyperintensity (WMH) volumes were measured from T2-weighted MRI images, as previously described (35). To account for individual differences in head size, estimated total intracranial volume was used to normalize WMH volumes.

### Statistical analysis

Participant characteristics are presented as the mean ± standard deviation. Shapiro–Wilk tests were used to evaluate the normality of age, lipids (TG, total cholesterol, HDL-C, non-HDL-C, LDL-C, and VLDL-C), and cerebral perfusion (GM CBF, WM CBF, and HBF). Pearson correlations and simple linear regressions were used to assess the associations between blood lipids and cerebral perfusion. Furthermore, these associations were examined by *APOE* status with heterozygotic or homozygotic ε4 individuals grouped together as “ε4 carriers” and individuals with a *APOE* genotype containing only ε2 or ε3 were grouped together as “non-ε4 carriers.” The group differences between ε4 carriers and non-ε4 carriers were evaluated using unpaired t-tests. Potential covariates including age, sex, cognitive function, p-tau 217, and WMH volumes were tested for interaction using multiple linear regressions. Statistical analyses and figures were generated using GraphPad Prism 8.0 (GraphPad Software) with significance set at *P* ≤ 0.05.

## Results

### Participant characteristics

Of the 65 participants enrolled, 47 participants were non-ε4 *APOE* carriers and 18 participants were either heterozygotic (n = 17) or homozygotic (n = 1) ε4 carriers. Participant characteristic variables represented by *APOE* status (ε4 carriers and ε4 noncarriers) were not significantly different between groups (*P* > 0.05 for all), except p-tau 217 was significantly higher in ε4 carriers than ε4 noncarriers (*P* < 0.001), as seen in Table 1. Within this group difference in p-tau 217, six ε4 carriers (33%) and four non-ε4 carriers (9%) had levels above ≥0.42 pg/ml, which is a binary reference for amyloid positivity (34). In all participants, age, p-tau 217, and WMH volume were negatively associated with GM CBF and HBF (range: r = −0.30 to −0.51; *P* = <0.001 to 0.029), but only WMH volume was negatively associated with WM CBF (r = −0.24; *P* = 0.026). These significant associations were independent of *APOE* status, except the age-related decline in HBF was significant in non-ε4 carriers (r = −0.41; *P* = 0.002), but not in ε4 carriers (r = −0.19; *P* = 0.226), and the association between WMH volume and WM CBF was significant in ε4 carriers (r = −0.57; *P* = 0.006), but not in non-ε4 carriers (r = −0.16; *P* = 0.147). No effects of sex or cognitive function on resting cerebral perfusion were observed.

**Table 1:** Participant characteristics (mean ± standard deviation)

*APOE* Genotype	ε4 Carriers (N = 18)	Non-ε4 Carriers (N = 47)	Combined (N = 65)
Age (years)	65 ± 10	62 ± 9	63 ± 9
Female sex–no. (%)	10 (56)	31 (66)	41 (63)
Male sex–no. (%)	8 (44)	16 (34)	24 (37)
Height (cm)	168.1 ± 11.3	167.7 ± 9.3	167.8 ± 9.7
Body mass (kg)	74.5 ± 14.1	73.8 ± 13.2	74.1 ± 13.4
Body mass index	26.4 ± 3.5	26.2 ± 3.7	26.2 ± 3.6
Systolic BP (mmHg)	119 ± 15	119 ± 12	119 ± 13
Diastolic BP (mmHg)	69 ± 11	71 ± 7	71 ± 8
Mean arterial BP (mmHg)	86 ± 11	87 ± 8	87 ± 9
Heart rate (bpm)	60 ± 11	64 ± 9	63 ± 10
HVLT-R trial 1 (no. correct)	6.1 ± 2.3	5.7 ± 1.8	5.8 ± 2.0
HVLT-R trial 2 (no. correct)	8.4 ± 2.2	8.2 ± 2.2	8.3 ± 2.2
HVLT-R trial 3 (no. correct)	8.6 ± 2.4	9.7 ± 2.0	9.4 ± 2.1
HVLT-R total recall (T-score)	44 ± 11	44 ± 11	44 ± 11
HVLT-R delay recall (no. correct)^a^	8.3 ± 4.1	8.0 ± 3.3	8.1 ± 3.4
HVLT-R delay recall (T-score)^a^	46 ± 14	44 ± 13	44 ± 13
P-tau 217 (pg/ml)	0.32 ± 0.14^b^	0.18 ± 0.14^b^	0.22 ± 0.15
Total intracranial volume (cm^3^)	1,527 ± 168	1,430 ± 219	1,457 ± 209
WMH volume (cm^3^)	7.2 ± 3.9	6.7 ± 5.6	6.8 ± 5.1

BP, Blood pressure; bpm, beats per minute; cm, centimeters; cm^3^, cubic centimeters; HVLT-R, Hopkins verbal learning test; km, kilograms; no., number; mmHg, millimeters of mercury; pg/ml, picogram per milliliter; WMH, white matter hyperintensity.

a Indicates ε4 carriers (N = 13), non-ε4 carriers (N = 43), and combined (N = 58).

b Indicates significant difference between groups (*P* < 0.001).

### Blood lipids and cerebral blood flow

Blood lipids and CBF values, separated by *APOE* status (ε4 carriers and ε4 noncarriers) were not significantly different between groups (*P* > 0.05 for all), but there was a trend for HDL-C to be higher in ε4 noncarriers than ε4 carriers (*P* = 0.054) as seen in Table 2.

**Table 2:** Participant blood lipids and resting cerebral perfusion (mean ± standard deviation)

*APOE* Genotype	ε4 Carriers (N = 18)	Non-ε4 Carriers (N = 47)	Combined (N = 65)	Reference interval
Triglycerides (mg/dl)	102 ± 43	98 ± 27	99 ± 32	0–149
Total cholesterol (mg/dl)	190 ± 28	197 ± 30	195 ± 29	100–199
LDL-C (mg/dl)	112 ± 28	112 ± 25	112 ± 25	0–99
VLDL-C (mg/dl)	19 ± 7	17 ± 5	18 ± 5	5–40
HDL-C (mg/dl)	59 ± 12	67 ± 19	65 ± 18	>39
Non-HDL-C (mg/dl)	130 ± 28	130 ± 25	130 ± 26	0–129
GM CBF (ml/100 g/min)	58.4 ± 18.2	63.0 ± 18.3	61.7 ± 18.3	40–70
WM CBF (ml/100 g/min)	34.0 ± 9.5	34.0 ± 9.2	34.0 ± 9.2	20–40
HBF (ml/100 g/min)	61.6 ± 16.1	64.4 ± 15.7	63.6 ± 15.7	50–70

CBF, cerebral blood flow; GM, gray matter; HBF, hippocampal blood flow; HDL-C, high-density lipoprotein cholesterol; LDL-C, Low-density lipoprotein cholesterol; VLDL-C, very-low-density lipoprotein cholesterol; WM, white matter; mg/dl, milligram per deciliter; ml/100 g/min, milliliters per 100 g per minute.

**Fig. 1:**
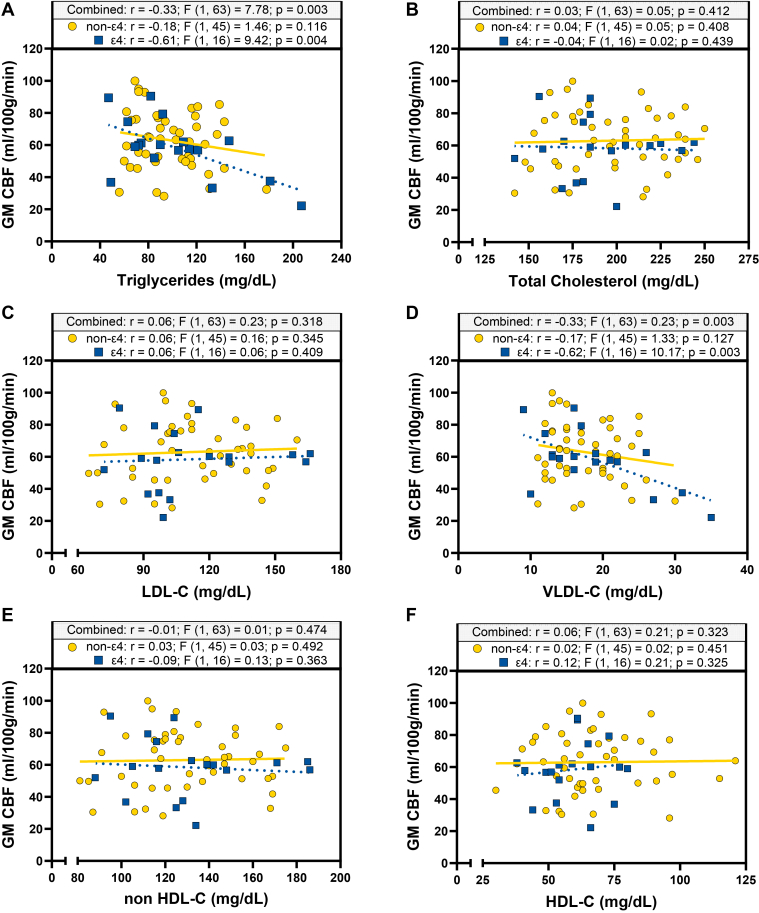
Association of triglycerides (A), total cholesterol (B), low-density lipoprotein cholesterol (LDL-C) (C), very-low-density lipoprotein cholesterol (VLDL-C) (D), non high-density lipoprotein cholesterol (non HDL-C) (E), HDL-C (F) with gray matter (GM) cerebral blood flow (CBF) using linear regressions.

**Fig. 2:**
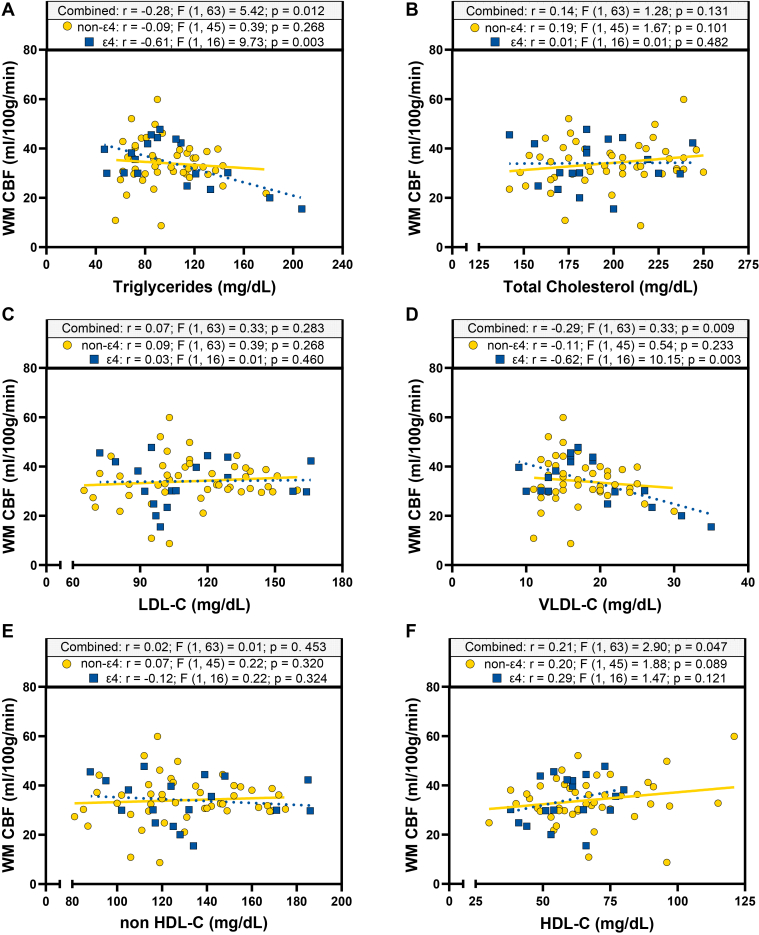
Association of triglycerides (A), total cholesterol (B), low-density lipoprotein cholesterol (LDL-C) (C), very-low-density lipoprotein cholesterol (VLDL-C) (D), non high-density lipoprotein cholesterol (non HDL-C) (E), HDL-C (F) with white matter (WM) cerebral blood flow (CBF) using linear regressions.

**Fig. 3:**
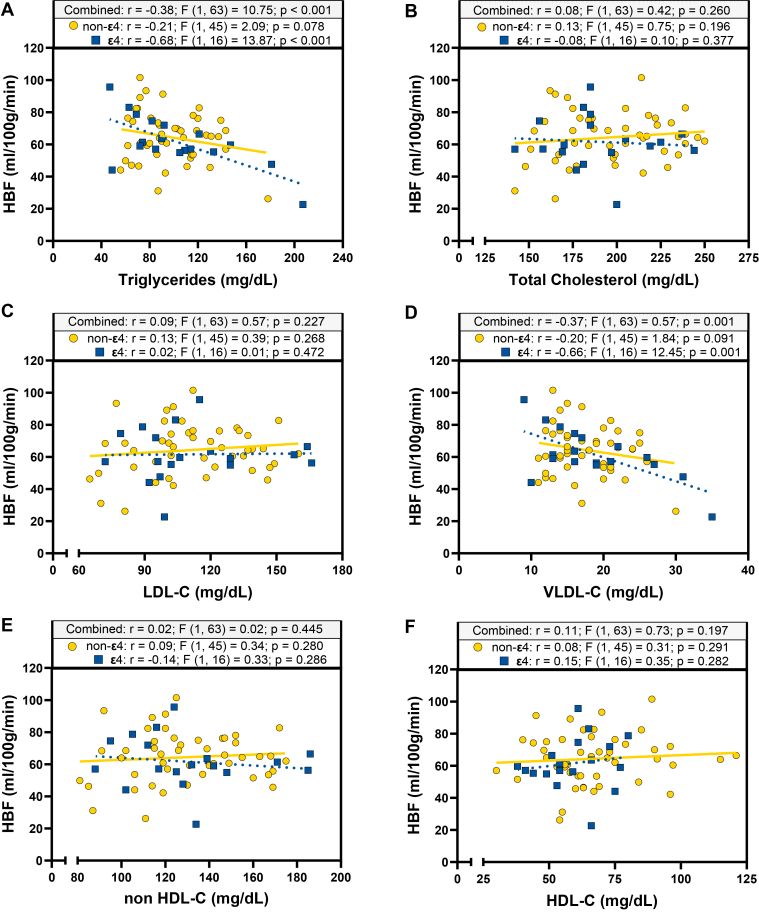
Association of triglycerides (A), total cholesterol (B), low-density lipoprotein cholesterol (LDL-C) (C), very-low-density lipoprotein cholesterol (VLDL-C) (D), non high-density lipoprotein cholesterol (non HDL-C) (E), HDL-C (F) with hippocampal blood flow (BBF) using linear regressions.

Serum TG was negatively associated with GM CBF: (r = −0.33; F (1, 63) = 7.78; *P* = 0.003), WM CBF: (r = −0.28; F (1, 63) = 5.42; *P* = 0.012), and HBF: (r = −0.38; F (1, 63) = 10.75; *P* < 0.001) in all participants. In all cases, the associations of TG were influenced by *APOE* genotype and only significant in ε4 carriers but not non-ε4 carriers with GM CBF, WM CBF, and HBF (Figs. 1A, 2A, 3A). No interactions between p-tau 217 and serum TG were observed on GM CBF (*P* = 0.338), WM CBF (*P* = 0.501), or HBF (*P* = 0.223).

Total cholesterol was not associated with GM CBF (r = 0.03; F (1, 63) = 0.05; *P* = 0.412), WM CBF (r = 0.14; F (1, 63) = 1.28; *P* = 0.131), or HBF (r = 0.08; F (1, 63) = 0.42; *P* = 0.260) in all participants. No differences by *APOE* status were observed for the associations of total cholesterol with GM CBF, WM CBF, and HBF (Figs. 1B, 2B, 3B).

LDL-C was not associated with GM CBF (r = 0.06; F (1, 63) = 0.23; *P* = 0.318), WM CBF (r = 0.09; F (1, 63) = 0.33; *P* = 0.283), or HBF (r = 0.09; F (1, 63) = 0.57; *P* = 0.227) in all participants. No differences by *APOE* status were observed for the associations of LDL-C with GM CBF, WM CBF, and HBF (Figs. 1C, 2C, 3C).

VLDL-C was negatively associated with resting GM CBF (r = −0.33; F (1, 63) = 0.23; *P* = 0.003), WM CBF (r = −0.29; F (1, 63) = 0.33; *P* = 0.009), and HBF (r = −0.37; F (1, 63) = 0.57; *P* = 0.001) in all participants. These associations of VLDL-C on resting cerebral prefusion were influenced by *APOE* genotype as all associations were significant in ε4 carriers, but not non-ε4 carriers, with GM CBF, WM CBF, and HBF (Figs. 1D, 2D, 3D). No interactions between p-tau 217 and VLDL-C were observed on GM CBF (*P* = 0.429), WM CBF (*P* = 0.413), or HBF (*P* = 0.279).

HDL-C was not associated with GM CBF (r = 0.06; F (1, 63) = 0.21; *P* = 0.323) or HBF (r = 0.11; F (1, 63) = 0.73; *P* = 0.197) but was significantly associated with WM CBF (r = 0.21; F (1, 63) = 2.90; *P* = 0.047) in all participants. No differences by *APOE* status were observed for the associations of HDL-C with GM CBF, WM CBF, and HBF (Figs. 1E, 2E, 3E).

Non-HDL-C was not associated with GM CBF (r = −0.01; F (1, 63) = 0.01; *P* = 0.474), WM CBF (r = 0.02; F (1, 63) = 0.01; *P* = 0. 453), or HBF (r = 0.02; F (1, 63) = 0.02; *P* = 0.445) in all participants. No differences by *APOE* status were observed for the associations of non-HDL-C with GM CBF, WM CBF, and HBF (Figs. 1F, 2F, 3F).

## Discussion

In this study, we assessed the associations between blood lipids and resting cerebral perfusion, stratified by *APOE* genotype, in a small group of healthy midlife and older adults. We found that serum TGs and VLDL-C were negatively associated with GM and WM CBF, and HBF, and HDL-C was positively associated with WM CBF. When stratified by *APOE* status, the observed negative associations were more pronounced in ε4 carriers than non-ε4 carriers, despite no significant group differences in lipids. Collectively, our findings suggest resting CBF is negatively affected by circulating blood lipids, particularly among ε4 carriers, and may partially explain the increased *APOE* genetic risk for AD pathology.

Our findings that serum TG was negatively associated with resting cerebral perfusion, including perfusion to regions such as the hippocampus, agrees with prior work (36, 37, 38). To our knowledge, we are the first to expand on these findings that VLDL-C was also negatively associated with resting cerebral perfusion. Since the primary lipid transported by VLDL-C is TG (39), it was not unexpected to find that both TG and VLDL-C were negatively associated with resting cerebral perfusion. Among the previous research on *APOE* status affecting cerebral perfusion (24, 25, 40, 41, 42, 43, 44), Wang and colleagues found that a poor cardiometabolic profile is related to the age-related reductions in cerebral perfusion that were dependent on *APOE* status (24). However, their analysis on blood lipids was limited to total cholesterol, thus the novelty in our current study is the inclusion of a blood lipid panel to determine associations on cerebral perfusion.

The exact mechanisms by which the ε4 allele of the *APOE* gene confers increased risk for AD remains less clear but appears to be a multifaceted modulation by nonmodifiable (i.e., age, sex, race, and ethnicity) and modifiable risk factors (45, 46, 47, 48). The *APOE* isoforms (ε2, ε3, and ε4) have distinct functions in regulating brain lipid transport and neuroinflammatory signaling, which may differentially regulate amyloid beta clearance, aggregation, and deposition in the brain (49). Plasma p-tau 217, a surrogate measure of amyloid beta presence in the brain (34), was an independent predictor of cerebral perfusion and measurably higher in ε4 carriers. However, in our relatively small sample size, there was no indication that p-tau 217 influenced the effects of blood lipids on reduced resting cerebral perfusion by *APOE* status. A potential upstream mechanism to explain how *APOE* status can influence resting cerebral perfusion is through disruptions in the blood-brain-barrier, which have been detected in ε4 carriers (50, 51). Serum TG can influence the regulation of insulin transport across the blood-brain-barrier (52, 53), and insulin is a known CBF modulator. However, additional research is needed to establish a causation between serum TG and cerebrovascular dysfunction leading to the onset of AD.

Our group has previously reported serum TG were associated with lower memory performance and hippocampal viscoelasticity, a measure of brain microstructural integrity assessed through magnetic resonance elastography (54). Although TG have been shown to independently affect cognitive function (55), even when controlling for *APOE* status (56), there remains a paucity of data regarding the influence of TG levels on cerebrovascular function. Some studies have correlated elevated midlife TG and increased AD risk later in life (57), while others have reported opposite results (58, 59). These discrepancies may be due to age differences among studies, as elevated triglycerides may constitute a more important risk factor for cognitive health prior to, as opposed to after, turning 65 years old (60). In a separate report, participants with higher lipid variability, or fluctuations in blood lipid levels over time, were shown to have a greater risk for developing AD compared to the absolute lipid value (61). Therefore, point measurements of blood TG may not optimally reflect an individual’s ability to maintain metabolic homeostasis, and more extensive characterizations of metabolic status may be required to fully understand how TG influence AD risk. Because TG naturally fluctuates with age, there may be varying effects on cerebrovascular health according to *APOE* status. This would underscore the importance of understanding individual variation as a contributing factor to promoting the risk of developing AD.

Contrary to our hypothesis, we did not find an association between total cholesterol or LDL-C and resting cerebral perfusion. This was surprising because of LDL-C known atherogenic effects (62, 63) and strong association with AD risk (64, 65). However, the lack of group differences in LDL-C between ε4 carriers and noncarriers, low number of clinically hyperlipidemic participants, and the relatively small sample size may have played a role. It is also possible that longitudinal changes in LDL-C are more important than cross-sectional correlations, as *APOE* polymorphisms are associated with longitudinal changes in total and LDL-C (12) or that variability in LDL-C affects CBF more than the absolute concentration, as previously described (66).

Beyond its direct role in influencing lipid homeostasis, there is emerging evidence that different *APOE* genotypes may contribute to neuroinflammation (67) by triggering an innate immune response and altered glial cell cytokine release (68, 69). Our laboratory has also shown that circulating T-lymphocytes (T cells) may modulate cerebrovascular function through their production of inflammatory cytokines and reactive oxygen species and that T-cell activation is directly induced by LDL-C (70, 71); however, the role of *APOE* was not explored in these studies. Many lipid lowering medications also exhibit anti-inflammatory properties (72, 73). While the efficacy of these drugs for lowering lipids is generally stronger in non-*ε4* carriers (74, 75), the effect *of APOE* on the anti-inflammatory effects of lipid lowering drugs requires additional research.

In contrast to other blood lipids, HDL-C was positively associated with WM CBF, but not GM CBF or HBF. Indeed, WM tissue exhibits vascular densities four to eight times lower than GM (76, 77). Although not observed in our cohort, HDL-C and the HDL-C/LDL-C ratio have been positively associated with WM microstructure and WMH volumes (78, 79). An explanation could be that our cohort WMH volumes were between the 50 and 75th percentile this age group (80), suggesting a mild burden of small vessel disease. The tendency that HDL was higher in non-*ε4* carriers than *ε4* carriers (*P* = 0.054) could be explained by our cohort being predominantly females, which did have higher HDL-C than males. However, we did not observe any sex differences in resting cerebral perfusion that would explain the association between HDL-C and WM CBF.

The strength of the current work includes an accurate and reliable measurement of resting cerebral perfusion using pCASL that can distinguish between GM, WM, and regional CBF, such as the hippocampus. Additionally, no participants were on lipid-lowering medications that would confound the observed associations. We included midlife and older (≥50 years old) adults in this study as they are vulnerable to increases in cardiometabolic risk factors for dementia. Among the 65 participants, our cohort of ε4 carriers (29%) and non-ε4 carriers (71%) was consistent with the *APOE* genotype distribution in the general population (9). All females in our study were at least 1-year postmenopause, which reduces the influence of sex hormones on cerebral perfusion outcomes. Future work should focus on longitudinal changes in blood lipids during the menopausal transition to determine whether *APOE* genotype influences these changes.

Our cohort of ε4 carriers and ε4 noncarriers was limited by a narrow range of blood lipids, as all but three participants had serum TG and VLDL-C within their normal preclinical ranges (<150 mg/dl and <30 mg/dl, respectively). Indeed, no participants were on any lipid-lowering medications (e.g., statins and fibrates) to control for the confounding effects of these drugs, as they can improve CBF in healthy adults with a familial history of AD (81) and cerebrovascular disease patients (82, 83, 84). A sensitivity analysis excluding participants with hyperlipidemia (two ε4 carriers and one non-ε4 carriers) showed the strength of the negative associations TG and VLDL-C had with cerebral perfusion were attenuated, although remained more pronounced in ε4 carriers. These findings suggest that elevated TG and VLDL-C are the high leverage data points that bias the significance of the negative associations between APOE status. To address this limitation, future directions should determine the influence of hyperlipidemia on cerebral perfusion by *APOE* status. Further, research that included apolipoproteins A1 and B would provide additional insight into the lipid/protein ratio that can influence cerebral perfusion. While our results focused on resting cerebral perfusion, future investigators can use dynamic measures of cerebrovascular health such as cerebrovascular reactivity to stimuli such as carbon dioxide, acetazolamide, post-prandial, or exercise (85, 86), which has yet to be scientifically studied.

In summary, our findings suggest serum TG and VLDL-C are negatively associated with resting cerebral perfusion while HDL-C is positively associated with WM blood flow in midlife and older adults. While the negative influence of TG and VLDL-C on resting cerebral blood flow may be more pronounced in ε4 carriers, future research should expand on this area to include patients with hyperlipidemia. Continued research in this area may advance personalized medicine for blood lipid management according to *APOE* status. In conclusion, blood lipids are an important modifiable cardiometabolic risk factor to maintain cerebrovascular function which may help mitigate future AD risk.

## Data availability

Data will be made available upon reasonable request.

## Conflicts of interest

The authors declare that the research was conducted in the absence of any commercial or financial relationships that could be construed as a potential conflict of interest.
